# Evidence for Restriction of Ancient Primate Gammaretroviruses by APOBEC3 but Not TRIM5α Proteins

**DOI:** 10.1371/journal.ppat.1000181

**Published:** 2008-10-17

**Authors:** David Perez-Caballero, Steven J. Soll, Paul D. Bieniasz

**Affiliations:** Aaron Diamond AIDS Research Center and Laboratory of Retrovirology, The Rockefeller University, New York, New York, United States of America; Northwestern University, United States of America

## Abstract

Because of evolutionary pressures imposed through episodic colonization by retroviruses, many mammals express factors, such as TRIM5α and APOBEC3 proteins, that directly restrict retroviral replication. TRIM5 and APOBEC restriction factors are most often studied in the context of modern primate lentiviruses, but it is likely that ancient retroviruses imposed the selective pressure that is evident in primate TRIM5 and APOBEC3 genes. Moreover, these antiretroviral factors have been shown to act against a variety of retroviruses, including gammaretroviruses. Endogenous retroviruses can provide a ‘fossil record’ of extinct retroviruses and perhaps evidence of ancient TRIM5 and APOBEC3 antiviral activity. Here, we investigate whether TRIM5 and APOBEC3 proteins restricted the replication of two groups of gammaretroviruses that were endogenized in the past few million years. These endogenous retroviruses appear quite widespread in the genomes of old world primates but failed to colonize the human germline. Our analyses suggest that TRIM5α proteins did not pose a major barrier to the cross-species transmission of these two families of gammaretroviruses, and did not contribute to their extinction. However, we uncovered extensive evidence for inactivation of ancient gammaretroviruses through the action of APOBEC3 cytidine deaminases. Interestingly, the identities of the cytidine deaminases responsible for inactivation appear to have varied in both a virus and host species–dependent manner. Overall, sequence analyses and reconstitution of ancient retroviruses from remnants that have been preserved in the genomes of modern organisms offer the opportunity to probe and potentially explain the evolutionary history of host defenses against retroviruses.

## Author Summary

Retroviruses integrate their genomes into host-cell DNA as an essential part of their replication cycle. If a retrovirus is integrated into a cell that becomes a germ line cell such as a sperm or an egg, then it may be inherited as an ‘endogenous’ retrovirus. In fact, endogenous retroviruses are extraordinarily common in mammalian DNA, constituting about 8% of human DNA. These endogenous retroviruses are mostly derived from ancient viruses that are now extinct. In this study, we recovered parts of two groups of extinct retroviruses, many strains of which became integrated into genomes of many nonhuman primates over the past few million years, but are absent from human DNA. We were able to generate infectious retroviruses by inserting a part of the extinct viruses into a modern retrovirus found in mice, and in so doing were able to functionally analyze properties of the extinct virus. Using a combination of these functional analyses, as well as sequence analysis, we obtained evidence that some rapidly evolving host defense molecules present in modern primates were able to inhibit the replication of these extinct viruses. Therefore, particular host defenses may have limited transmission of ancient retroviruses between species and perhaps contributed to their extinction.

## Introduction

Retroviruses integrate their genomes into host-cell DNA as an essential part of their replication cycle. If a provirus is integrated into the germ line or its progenitors, then it may be inherited in a Mendelian manner as an ‘endogenous’ retrovirus. In fact, endogenous retroviruses have accumulated over time in the genomes of many organisms and are extraordinarily common in mammalian genomes, including that of humans [1],[2]. Perhaps because of these episodic insults by retroviruses, mammals express proteins, such as TRIM5α and the APOBEC3 family of proteins, that directly inhibit retroviral replication [3],[4]. Indeed, TRIM5α is responsible for a post-entry restriction of a variety of retroviruses in many primate species through the interaction with incoming retroviral capsids [4]–[8]. Additionally, the APOBEC3 proteins are cytidine deaminases that act primarily by infiltrating retroviral particles and thereafter catalysing cytidine deamination of single-stranded retroviral cDNA during reverse transcription, thus inhibiting viral replication [9]–[16]. Evidence for strong selection pressure on these antiviral factors comes from several observations. First, the APOBEC3 gene family has expanded from one gene in mice to seven genes in primates [17]. Second, sequence analyses of primate APOBEC3 and TRIM5 genes, reveal that they have been under strong diversifying (positive) pressure since the divergence of old and new world monkeys 33 million years ago [18]–[20] as well as balancing selection in certain species [21]. Third, there is a striking example of convergent evolution at the TRIM5 locus where a new hybrid TRIM5/cyclophilin gene has independently arisen via retrotransposition and has been selected in two distinct primate species [22]–[27]. Although TRIM5 and APOBEC restriction factors are most often studied in the context of modern primate lentiviruses, it seems likely that these viruses emerged too recently to explain the selective pressure that was evidently imposed on primate TRIM5 and APOBEC3 genes [18]–[20],[28],[29]. Rather, it is likely that APOBEC3 and TRIM5 evolved to combat ancient retroviruses, long before the appearance of primate lentiviruses.

Endogenous retroviruses can provide us with a “fossil record” of extinct retroviruses and perhaps evidence of ancient TRIM5 and APOBEC3 antiviral activity. Moreover, this record can be accessed in a nearly complete form as a result of recent genome sequencing and annotation efforts. In this study, we examined two families of endogenous gammaretroviruses (ERVs) that are relatives of murine leukemia virus (MLV) and are present in endogenous form in the genomes of apes and old world monkeys; multiple copies are present in the genome sequences of chimpanzees and rhesus macaques. Curiously, these same two families of MLV-related retroviruses are absent from the human genome [1],[30] for unknown reasons, while horizontal transfers, endogenization and extinction of these two families of viruses appears to have occurred during the past few million years in several other old world primate species. It is possible that antiretroviral factors such as TRIM5α and APOBEC3 proteins prevented cross-species transmission and perhaps even contributed to the apparent extinction of gammaretroviruses in many primate species. In this regard, it has recently been reported that the TRIM5 protein of humans is active against an ancestral form of one of the endogenous gammaretroviruses examined here (CERV1, otherwise referred to as ptERV1) [31].

By reconstituting a number of infectious chimeric retroviruses bearing capsid sequences from extinct primate gammaretroviruses, and by examining ‘fossilized’ gammaretroviral sequences, we investigated whether TRIM5 and APOBEC3 proteins restricted the replication of extinct gammaretroviruses that appear quite widespread in the genomes of old world primates but failed to colonize the human germline. Our analyses do not support the conclusion that TRIM5α proteins presented a major barrier to the cross-species transmission of two families of gammaretroviruses that were endogenized over the past few million years, or contributed to their extinction. However, we uncovered extensive evidence for inactivation of primate gammaretroviruses through the action of APOBEC3 cytidine deaminases. Interestingly, the identities of the cytidine deaminases responsible for inactivation of endogenous gammaretroviruses appear to have varied in both a virus and host species-dependent manner.

## Results

To investigate the potential contribution of APOBEC3 and TRIM5α proteins to the host range limitation and/or extinction of the two endogenized groups of primate gammaretroviruses, we retrieved and analyzed all unique capsid N-terminal domain (CA-NTD) sequences from chimpanzee endogenous retroviruses-1 and -2 (CERV1 and CERV2) as well as from closely related endogenous retroviruses in rhesus macaques (RhERV1 and RhERV2). For comparison, a set of related gammaretroviral sequences in mice, corresponding to endogenous murine leukemia virus (enMLV) were also analyzed. The sequences were retrieved by blast searching the nearly complete *Pan troglodytes*, *Macaca mulatta* and *Mus musculus* genome sequence databases, and group specific consensus sequences were computed.

**Figure 1:**
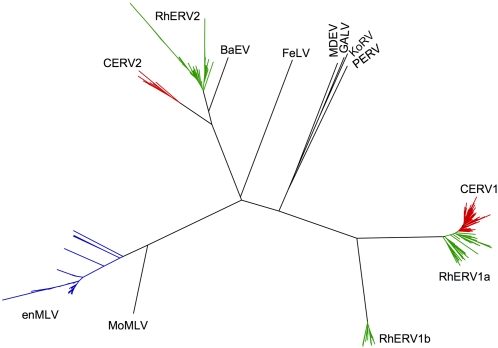
Phylogenetic tree illustrating the relationship between endogenous primate gammaretroviral capsid NTDs and other gammaretroviral CA-NTDs. A sequence alignment, and phylogeny of all capsid NTDs from CERV1, CERV2, RhERV1a, RhERV1b, RhERV2, and enMLV and various prototype gammaretroviruses was generated using ClustalX software. The tree diagram was generated and edited using FigTree software.

A phylogenetic tree illustrating the relationships among the CA-NTD sequences from these and other prototype gammaretroviruses is shown in Fig. 1. Eighty-five unique CERV1 CA-NTD sequences, forming a single clade, were found in the chimpanzee genome sequence and two clades of CA-NTDs, that were closely related to CERV-1, termed RhERV1a and RhERV1b, containing 48 and 28 unique members, respectively, were present in the rhesus macaque genome sequence database. RhERV1a was clearly more closely related to CERV-1 than RhERV1b. Another family of endogenous gammaretroviruses included the chimpanzee derived CERV2, for which only 10 unique CA-NTD sequences are present in chimpanzee DNA and RhERV2, for which 44 unique sequences were present in Rhesus macaque DNA. CERV2 and RhERV2 CA-NTD sequesces are closely related to the CA-NTD of Baboon endogenous retrovirus (BaEV). For comparative purposes, forty-one unique CA-NTD sequences from endogenized MLV (enMLV), some variants of which have recently been shown to bear mutations characteristic of APOBEC3-induced mutation [32], were also recovered from the mouse genome database (Fig. 1).

### MLV chimeric viruses containing primate and mouse endogenous gammaretroviral CA-NTDs are generally resistant to TRIM5α proteins

To determine whether inhibition by species-specific variants of TRIM5α proteins could have limited cross species transmission of these gammaretroviruses, or could have been responsible for their extinction in nonhuman old world primates, we constructed libraries of recombinant MLVs encoding chimeric CA proteins. Specifically, primate CA-NTDs were amplified from chimpanzee and rhesus macaque genomic DNA and introduced into an MLV Gag-Pol expression plasmid in place of the native MLV CA-NTD. Only the NTD of capsid was introduced into the recombinant MLVs because: (i) its structure is well conserved in diverse retroviruses and it contains all of the known determinants for restriction by TRIM5α [5]–[8],[33],[34] and (ii) confining the analysis to this smaller CA domain meant that a reasonable proportion of the amplified natural variants would be devoid of stop codons and, therefore, potentially amenable to functional analysis. For comparative purposes, similar libraries encoding enMLV CA-NTDs were generated.

The libraries of recombinant retroviruses contained many hundreds of members that were subjected to the functional analyses outlined below. However, sequence analyses of these libraries revealed many discrepancies between the amplified sequences and sequences present in the genome databases. In principle, these discrepancies could arise as a result of (i) natural variation in endogenous CA sequences, (ii) sequencing errors in the genome databases, (iii) recombination between closely related amplicons during PCR/cloning and (iv) Taq polymerase errors. Thus, the ensuing analyses exclude CA-NTD sequences that did not perfectly match a variant that was present in the sequence databases. However, the conclusions described below were precisely the same when large numbers of additional CA-NTD sequences that did not perfectly match a database CA-NTD sequence, were also analyzed (data not shown).

**Figure 2:**
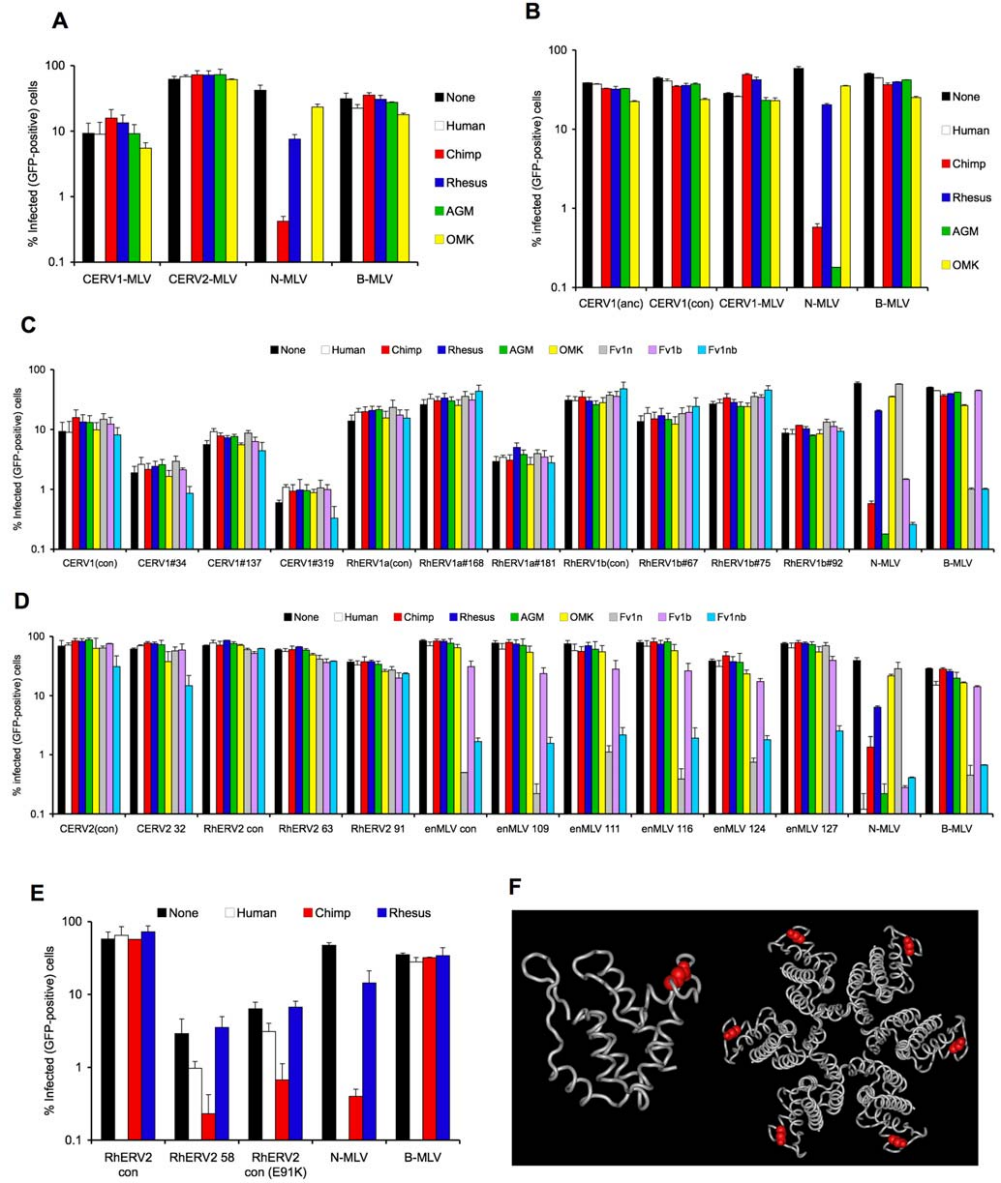
Resistance of chimeric MLVs encoding primate gammaretroviral NTD to primate TRIM5 proteins. A) MLV chimeric viruses encoding CA-NTD consensus sequence from CERV1 and CERV2 were used to infect MDTF cells expressing human, chimpanzee, rhesus and African green monkey TRIM5α or Owl Monkey TRIMCyp proteins. GFP positive cells were quantified by FACS (Guava EasyCyte) 48 h after infection. N-MLV and B-MLV were used as controls. B) MLV chimeric viruses containing an entire ancestral CERV1 capsid sequence (CERV-1(anc)), an entire consensus CERV1 capsid sequence (CERV1(con)) or a consensus CERV1 CA-NTD fused to an MLV CTD (as in A) were used to infect MDTF cells expressing the indicated TRIM5 proteins. N-MLV and B-MLV were used as controls and infection was evaluated as in A). C) MLV-chimeric viruses encoding natural variant CA-NTDs from CERV1, RhERV1a, RhERV1b, were used to infect MDTF cells expressing various TRIM5 and Fv1 proteins. D) MLV-chimeric viruses encoding natural variant CA-NTDs from CERV2, RhERV2 and enMLV were tested as in A). E) MLV chimeric viruses encoding a consensus RhERV2 CA-NTD, or a natural variant CA-NTDs (RhERV2 58) that includes the E91K variant and a point mutant of the consensus RhERV2 CA-NTD (E91K) mutant were tested as in A). F) Representation of the MLV CA-NTD with the positional equivalent of the RhERV2 E91 residue colored red. The left picture shows a side view of the CA-NTD monomer (outer surface of the capsid at the top) while the right picture shows a view of a single CA-NTD hexamer, viewed from the outside of the capsid.

To validate our approaches to test the TRIM5 sensitivity of extinct primate gammaretroviruses, we first analyzed MLV-derivatives containing CA-NTD variants from the chimpanzee endogenous gammaretroviruses (CERV1 and CERV2) that precisely matched the consensus sequence for each virus (termed CERV1-MLV and CERV2-MLV, see Dataset S1). Both of these recombinant viruses were quite infectious, yielding ∼10^4^ infectious units/ml of unconcentrated viral supernatant (Fig. 2A). Notably, however, both CERV1-MLV and CERV2-MLV behaved like B-tropic MLV in that they were entirely resistant to all of the TRIM5 proteins tested. Conversely, a control virus, N-tropic MLV, was strongly inhibited by human, chimpanzee and African green monkey TRIM5α proteins (Fig. 2A). These results contrast with those obtained using a CERV1-MLV chimeric virus previously described by Kaiser et al. that is significantly less infectious than the CERV1-MLV chimera described herein, but was reported to be sensitive to inhibition by human and chimpanzee TRIM5α [31]. However, Kaiser et al. introduced ancestral (rather than consensus) sequences and, morever, included the full-length capsid (NTD and CTD) as well as the flanking p12 domain from CERV1 in an MLV Gag-Pol plasmid. Our consensus CERV1 CA-NTD sequence differs at one amino acid position from the ancestral CERV1 CA-NTD sequence used by Kaiser et al. (R35 in the consensus sequence CERV1, is replaced by Q35 in the ancestral CERV1 CA). Therefore, we constructed chimeric MLV-based viruses containing a full-length CERV-1 capsid (both NTD and CTD) based on our own consensus CERV-1 NTD sequence, or the ancestral NTD CERV-1 sequence (Dataset S1). These recombinant viruses were highly infectious (yielding ∼10^5^ IU/ml) but, importantly, neither were sensitive to any TRIM5α protein tested (Fig. 2B). Thus, these data argue that CERV1 capsids are not sensitive to either human or chimpanzee TRIM5α, and indicates that our strategy in which the CA-NTDs were introduced into MLV results in capsids that accurately recapitulate the TRIM5α (in)sensitivity of the entire ERV capsid.

A broader analysis of MLV recombinants (CERV1-MLV, CERV2-MLV, RhERV1a-MLV, RhERV1b-MLV, RhERV2-MLV and enMLV-MLV) containing respective consensus ERV CA-NTD sequences or naturally occurring variants (Dataset S1), revealed that almost all were completely resistant to the collection of primate TRIM5α proteins, and also to the murine restriction factors Fv1^N^, Fv1^B^ and Fv1^NB^ (Fig. 2C, D). All but one enMLV CA-NTD recovered from NIH3T3 cell DNA displayed B-tropism (sensitivity to Fv1^N^ and Fv1^NB^) (Fig. 2B and C), while one clone was NB-tropic (Fig. 2D). Only one recombinant virus containing a primate gammaretrovirus CA-NTD, a RhERV2-MLV clone, that contained 4 mutations relative to consensus RhERV-2 sequence, exhibited any sensitivity to a TRIM5 protein. Specifically, infection by this RhERV2-MLV variant was inhibited approximately 10-fold by chimpanzee TRIM5α and marginally by human TRIM5α. (Fig. 2E). This clone had a mutation (E91K) that is present in ∼20% of the endogenized RhERV2 CA-NTD sequences and, using the MLV CA-NTD as a model [33], is predicted to be exposed on the surface of the mature hexameric capsid lattice (Fig. 2F). Notably, introducing the E91K mutation into the consensus RhERV2-MLV chimera recapitulated the sensitivity pattern observed with the original, naturally occurring RhERV2 CA-NTD (Fig. 2E), thereby identifying a novel determinant of retroviral sensitivity to TRIM5α proteins. However, this was the sole instance of sensitivity to a TRIM5α protein that was present in the libraries of recombinant MLVs containing CA-NTDs from endogenous chimpanzee, rhesus macaque and mouse gammaretroviruses.

### Analysis of sequence diversity in CA-NTD sequences from endogenous chimpanzee and rhesus macaque gammaretroviruses

**Figure 3:**
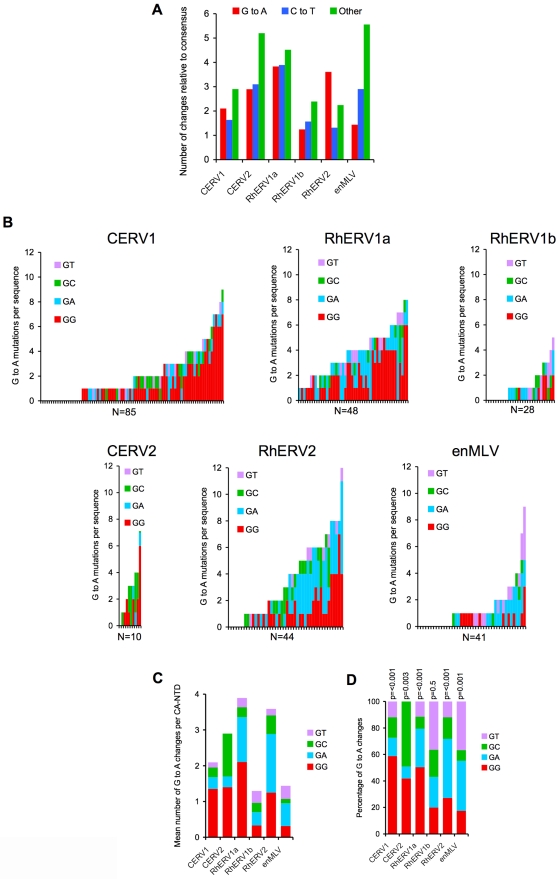
Analysis of endogenous primate gammaretrovirus CA-NTD sequences. A) The mean number of G to A, C to T, and all other changes (pooled) per CA NTD sequence, relative to each consensus sequence is shown. B) The numbers of G to A mutations for each individual CA-NTD recovered from sequence databases is plotted as a bar graph (one bar for each provirus) and color-coded according to dinucleotide context (i.e. the nucleotide in the +1 position relative to the G to A change; red = GG to AG, cyan = GA to AA, green = GC to AC, magenta = GT to AT). The CA NTD sequences are arranged from left to right in order of increasing numbers of total G to A changes. C) The mean number of G to A changes per CA-NTD sequence, relative to the consensus, is plotted for pooled data for each virus species and color coded according to dinucleotide context as in B. D) The percentage of G to A changes in each dinucleotide context relative to consensus sequence is plotted, and normalized according to the dinucleotide composition of the species-specific consensus sequences. The p-values for deviation from a random distribution were calculated using the chi-squared test.

Another major antiretroviral activity in primates that can inhibit a number of retroviruses consists of an array of APOBEC3 cytidine deaminases. To investigate whether these might have contributed to the apparent extinction of gammaretroviruses in chimpanzees and rhesus macaques, we first compared the CA-NTD sequences recovered from database searches with corresponding virus-species consensus sequences. Overall, this analysis revealed a varying, but relatively modest, degree of divergence, typically <3% for each individual CA-NTD relative to each virus-specific consensus sequence. However, the nature of the mutations that distinguished each group of CA-NTD sequences from their respective consensus varied. In particular, in several cases, there was a striking excess of changes that could potentially be attributable to cytidine deamination (G to A and C to T changes) (Fig. 3A). Moreover, different patterns of mutation were observed in viruses from the same species. For example, in RhERV1a and RhERV1b sequences, G to A and C to T changes occurred at approximately equal frequencies, but less frequently than pooled ‘other’ changes (Fig. 3A). Conversely, in RhERV2 database entries, G to A changes were clearly the most frequent mutation and comprised approximately one half of all the changes relative to the RhERV2 consensus CA-NTD sequence (Fig. 3A). In general, G to A mutations in endogenous primate gammaretroviruses comprised a significantly higher fraction of the total mutations than was the case in endogenous MLVs (Fig. 3A).

G to A and C to T mutations are the most common mutations found in mammalian genomes. Therefore, the excessive G to A and C to T mutations observed in endogenous gammaretrovirus sequences could be the result of spontaneous, post integration mutation, or could reflect encounters with specific antiretroviral cytidine deaminases. If these endogenous retroviruses were indeed mutated by APOBEC3 enzymes, then biases in the dinucleotide contexts in which G to A mutations are observed should be evident. For example, APOBEC3G induced mutation results in the biased appearance of plus strand G to A changes in the context of GG dinucleotides (i.e. GG to AG mutations). Conversely, several other APOBEC3 proteins, including primate APOBEC3F, 3B, 3H and murine APOBEC3 proteins preferentially induce GA to AA mutations [9], [12]–[14],[16],[35]. Only rarely have APOBEC enzymes, specifically APOBEC1, been reported to induce plus strand C to T mutations in retroviruses, by targeting viral genomic RNA [36].

Therefore, as a preliminary indication of whether these extinguished primate gammaretroviruses had encountered antiviral cytidine deaminases we analyzed all the recovered CA NTD sequences for specific dinucleotide context biases accompanying G to A and, as a control, C to T changes. Because the size of the CA-NTD encoding genomic region analyzed (∼400 nucleotides), and consequently the number of mutations observed in each individual CA-NTD, was rather small, we inspected the pooled CA-NTD sequence data (Fig. 3C) as well as the individual CA-NTD sequences (Fig. 3B) for each virus species. There was significant variation in the burden of G to A mutations among individual CA-NTDs, with some completely lacking G to A mutations while other contained up to 12 G to A mutations in the ∼400 nucleotide CA-NTD encoding sequence (Fig. 3B). Moreover, striking and variable biases in the patterns of G to A mutations were observed. For example, in CERV1 CA-NTDs, G to A changes occurred primarily in the context of GG dinucleotides (Fig. 3B, C). This bias was statistically significant, and was maintained when corrected for the frequency with which each GN dinucleotide occurred in the consensus sequence (Fig. 3D). In CERV2 CA-NTDs, the occurrence of G to A changes was biased toward both GG and GC dinucleotides. However, in this case, the overall numbers of G to A changes were small (Fig. 3B) because only 10 CERV2 proviruses are present in chimpanzee DNA. Interestingly, the closely related rhesus macaque endogenous gammaretroviruses RhERV1a, RhERV1b and RhERV2 exhibited a different mutational bias as compared to CERV1 and CERV2 chimpanzee counterparts: G to A mutations in RhERV1a and RhERV2 occurred frequently at both GG and GA dinucleotides (Fig. 3B, C), again these biases were highly statistically significant (Fig. 3D). In contrast, RhERV1b CA NTDs were quite different in that they had relatively few G to A mutations and their occurrence was comparatively unbiased with respect to dinucleotide context (Fig. 3B, C, D).

### Confirmation of mutational biases in endogenous gammaretroviruses

These data suggested that some endogenous proviruses may well have encountered antiviral cytidine deaminases. Therefore, to confirm and extend these analyses, we asked if similar patterns of mutation could be observed in another region of the endogenous proviruses. In particular we analyzed Env sequences, which are obviously longer than CA-NTD sequences and permit a more robust estimate of context-specific mutation frequencies in individual proviruses. Retrieval and analysis of the endogenous gammaretroviral Env sequences revealed that their phylogenetic relationships to each other and to other gammaretroviruses were similar to those observed using CA-NTD sequences (Fig. S1). The exception to this was BaEV, which was closely related to CERV2 and RhERV2 in CA, but not in Env, presumably as a result of recombination between ancestral viruses.

**Figure 4:**
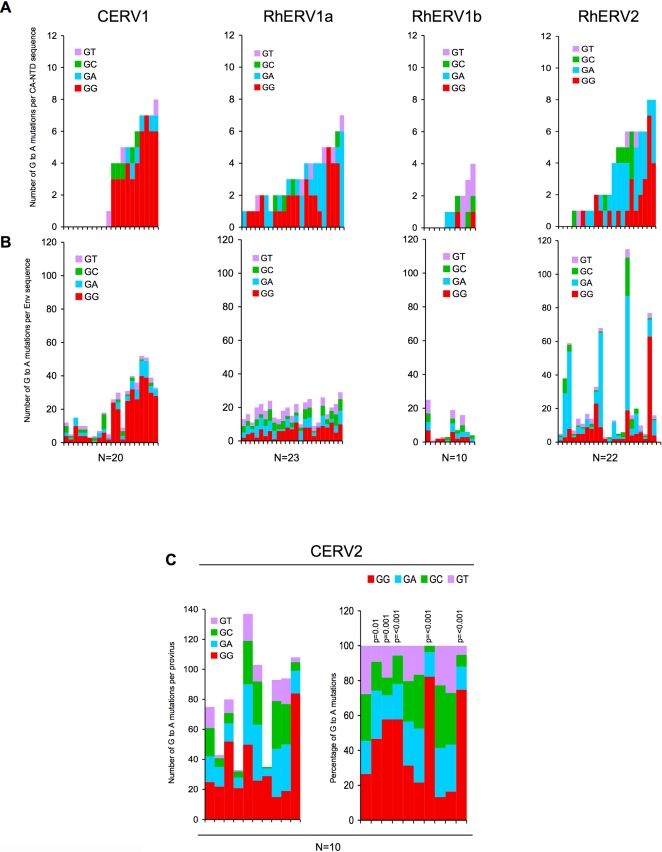
Analysis of G to A changes in CA-NTD and Env sequences in primate gamaretroviruses. A) CA-NTD sequences from the primate gamaretroviruses were selected according to whether an unambiguously linked Env sequence could be retrieved from the genome database. In the case of CERV1, ten sequences that lacked G to A changes and ten sequences that had four or more G to A changes were selected. For each virus species, each sequence is plotted as a bar graph (one bar for each provirus) and color-coded according to dinucleotide context (ie the nucleotide in the +1 position relative to each G to A change; red = GG to AG, cyan = GA to AA, green = GC to AC, magenta = GT to AT). The CA NTD sequences are arranged from left to right in order of increasing numbers of total G to A changes in CA-NTD sequences (upper panels). B) The same analysis was performed for the linked env sequences (lower panels), which are arranged in the same order (left to right) according to the linked CA-NTD sequence. C) All ten CERV2 proviral sequences were analyzed and the dinucleotide context in which G to A mutations occurred is plotted and color-coded as in A (left) panel. Additionally, The percentage of G to A changes in each dinucleotide context relative to consensus sequence is plotted, and normalized according to the dinucleotide composition of the consensus sequences (right panel). The p-values for deviation from a random distribution were calculated using the chi-squared test. Proviruses 1, 4, 6 and 7 are complete.

Because there are large numbers of CERV-1 proviruses in chimpanzee DNA, and the genome database is comparatively complete, we retrieved 10 individual Env sequences linked to CA-NTDs that had 1 or fewer G to A mutations and 10 Env sequences linked to CA-NTDs that contained 4 or more G to A mutations. Overall, there was a good correlation between the CA-NTD and Env sequences in terms of the burden and character of the G to A mutations (Fig. 4 A, B). Specifically, the occurrence of GG to AG mutations in a given CERV1 CA-NTD strongly predicted the occurrence of the same type of mutation in the linked Env sequence. Indeed, there was only one exception to this finding among the 20 CERV-1 sequences analyzed, in which a CA-NTD bearing three GG to AG and one GC to AC mutation was linked to an Env sequence containing a light burden of G to A changes (Fig. 4 A, B).

Because there are only ten CERV2 proviruses in the chimpanzee genome (and only four of them are complete, with others ranging in size from 2.6 to 7.7 Kb) we took a different approach to confirm or refute the notion that they contained excessive or biased G to A mutations. Specifically, we analyzed all of the available sequence for all ten proviruses. In part because the CERV2 proviruses were intrinsically divergent, and differed in length, the absolute numbers of G to A changes as compared to the consensus sequence was quite variable among them (Fig. 4C). However, when the character of the G to A mutations was examined, five of the 10 proviruses exhibited a clear, statistically significant, excess of GG to AG mutations, as compared to overall G to A changes (Fig. 4C). Thus, the patterns of nucleotide substitutions strongly suggested that both groups of endogenous gammaretroviruses that integrated into the chimpanzee genome in the past few million years frequently encountered the only mutator known to preferentially induce GG to AG mutations, namely APOBEC3G, prior to or during endogenization.

The coverage of the rhesus macaque reference genome database is less complete than that of the chimpanzee genome, so only 23, 10 and 22 envelope sequences that were unambiguously linked to CA-NTDs could be retrieved for RhERV1a, RhERV1b and RhERV2 respectively. Moreover, in the case of RhERV2, the CA-NTD linked Env sequences were distributed among two distinguishable subgroups (Fig. S1) and, therefore, a separate Env consensus sequence was deduced for each group. Surprisingly, in RhERV1a, the nature and burden of G to A changes in Env sequences was not well predicted by the findings in the CA-NTD sequences. Indeed, the preferential occurrence of G to A changes in Env in the context of GG and GA dinucleotides was marginal, and clearly less pronounced than in the CA-NTD sequences (Fig. 4, A, B). The reasons for this discordance are unclear and discussed below. However, for both RhERV1b and RhERV2 the analysis of Env sequences corroborated the results obtained using CA-NTDs (Fig. 4A, B). Specifically, the numbers of G to A changes in RhERV1b Env, like CA-NTD sequences, were modest and unbiased with respect to dinucleotide context, while in RhERV2, there was a clear excess of both GG to AG and GA to AA mutations in both CA-NTD and Env sequences. (Fig. 4A, B). However, while there was concordance with respect to overall context bias of G to A mutations present in RERV2 CA-NTD and Env sequences, inspection of individual linked CA-NTD and Env sequences revealed that the burden and character of G to A mutations was variable. Specifically, several Env sequences bore GA to AA hypermutation while two other Env sequences bore predominantly GG to AG hypermutation. Additionally, the degree to which CA-NTDs and Env sequences were mutated in individual proviruses did not always correlate; in some cases hypermutated RhERV2 Env sequences were linked to CA-NTD sequences that bore relatively few G to A changes (Fig. 4A, B). These results, and the discordant findings with respect to G to A mutation in RhERV1a CA-NTD versus Env sequences suggest that different regions of the genome can be mutated at different frequencies (a known characteristic of APOBE3G induced mutation). Alternatively it is conceivable thatrecombination between hypermutated and nonhypermutated viral genomes can occur prior to or during deposition in the germ line. Indeed, inspection of several complete or nearly complete RhERV2 proviruses (Fig. S2) revealed clear variation in the extent of G to A mutation across the viral genome in some (but not all) proviruses. Notably, an obvious 5′ to 3′ gradient of increasing mutation intensity was present in some examples bearing excessive G to A mutations, as has previously been reported in hypermutated HIV-1 genomes [35],[37].

### Biased patterns of G to A mutation in primate ERVs are not explained by spontaneous cytidine deamination

The chimpanzee and rhesus gammaretroviral CA-NTD and Env sequences also contained a striking excess of plus strand C to T changes relative to their respective consensus sequences (Fig. 3A). Potentially, the high rate of C to T mutations in CA-NTD sequences could be the result of spontaneous cytidine deamination after integration of the provirus into the germ line. Correspondingly, excessive plus strand G to A changes could be a reflection of spontaneous cytidine deamination events on the minus strand. In contrast to APOBEC3-mediated deamination/mutation events, whose frequency is profoundly influenced by the identity of the nucleotide in the −1 position relative to the deaminated cytidine, mutations that arise as a result of spontaneous cytidine deamination are influenced by the nucleotide in the +1 position. Specifically, the most common spontaneous cytidine deamination induced mutation occurs in the context of CG dinucleotides, (i.e. G in the +1 position). This results in CG to TG or CG to CA plus strand mutations, depending on whether CG dinucleotides on the plus or minus strand are deaminated, respectively [38],[39]. Therefore, to account for this potential source of “noise”, we counted and categorized all G to A and C to T changes for both CA-NTD and Env sequences before and after exclusion of CG dinucleotides in the minus and plus strand respectively (Fig. S3, S4). Notably, the biases that were associated with plus strand G to A mutations (equivalent to minus strand C to T mutations) were not evident in a similar analysis of plus strand C to T mutations. Moreover, exclusion of C to T mutations that occurred in the context of CG dinucleotides on the plus or minus strands, reduced the overall number plus strand G to A and C to T mutations but did not affect conclusions with respect to the dinucleotide context bias associated with G to A mutations (Fig. S3, S4).

Notably, the relative extent of G to A as compared to C to T mutations varied among individual proviruses of a given virus group as well as between virus groups. This was most evident in the analysis of Env sequences (or complete proviral sequences in the case of CERV2), because the longer length of sequence analyzed permitted a more robust estimate of G to A and C to T mutation frequencies (Fig. S4, S5). For many of the individual CERV1, CERV2, and RhERV2 proviruses, the frequency of G to A mutations greatly exceeded the frequency of C to T mutations (Fig. S4A, C, Fig. S5), and in proviruses where this was the case, the excessive G to A mutation was invariably in the context of GG or GA dinucleotides, and remained evident when CG dinucleotides were purged from the analysis (Fig. S4B, D, Fig. S5). Conversely, RhERV1a and RhERV1b Env sequences exhibited broadly similar levels of comparatively unbiased G to A and C to T mutation (Fig. S4) suggesting that the frequency of APOBEC3-induced mutation was low compared to that from other sources. A comparision with endogenous MLVs revealed a similar situation to that in primate gammaretroviruses, and as was previously reported, two out of the three groups of endogenous MLVs (Pmv, Mpv, but not Xmv) exhibited higher levels of G to A than C to T mutations, and this excess in G to A mutations was associated with similar context biases [32]. Overall, clear dinucleotide context biases were observed in each situation where G to A mutations clearly outnumbered C to T mutations.

Thus, the excessive and context-biased G to A mutations that are present in some of the groups of primate endogenous gammaretroviruses cannot be explained by spontaneous cytidine deamination. Rather, the analysis of both CA-NTD sequences, and linked envelope sequences, provides robust support for the hypothesis that APOBEC3G in chimpanzees, and a combination of APOBEC3G and other APOBEC3 proteins in rhesus macaques, extensively mutated gammaretroviral genomes prior to or during their endogenization.

### Effects of cytidine deamination on extinct primate gammaretrovirus CA-NTD function

**Figure 5:**
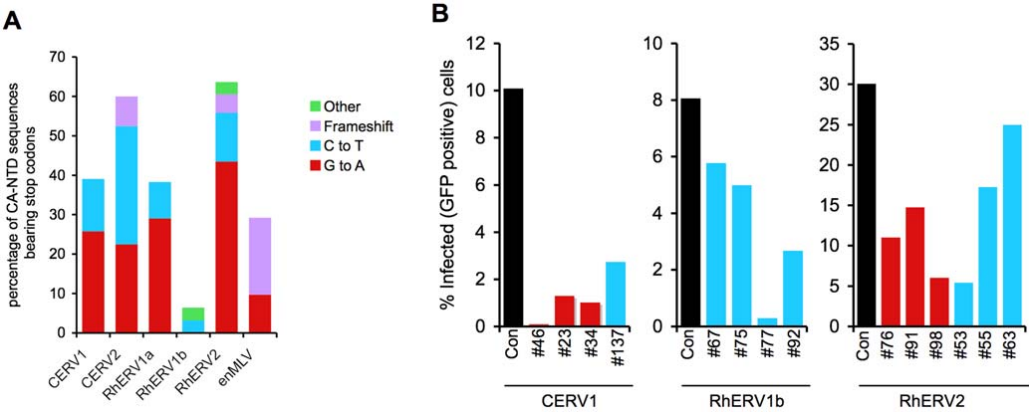
Effects of cytidine deamination on CA-NTD function. A) the percentage of translation termination codons generated by G to A, C to T, frameshift and other mutations in endogenous primate and mouse gammaretroviral CA-NTDs. The percentage of all CA-NTD sequences with stop codons in CERV1, CERV2, RhERV1a, RhERV1b, RhERV2, and enMLV CA-NTDs is plotted and the colored subdivision indicate the proportions that are attributable to each type of mutation. B) Effect of single G to A and C to T missense mutations on the infectivity of chimeric MLVs bearing CA-NTDs from primate endogenous retroviruses. Consensus capsid NTDs from the indicated virus species, and variants bearing single naturally occurring G to A mutations (red) or C to T mutations (cyan) were introduced into chimeric MLV GagPol and infectivity measured as in Fig. 1. Note that all three G to A mutations in CERV1 (#46, #23, #34) occurred in the context of GG dinucleotides, while the mutations in RhERV1a and RhERV2 were all in the context of CG dinucleotides on the plus strand (blue) or minus strand (red).

Inspection of the endogenous primate gammaretroviral CA-NTD sequences revealed that, with the exception of RhERV1b CA-NTD sequences, many harboured stop codons (Fig. 5A). Strikingly, a large fraction of the stop codons were generated by G to A mutations (Fig. 5A). C to T changes resulted in stop codons somewhat less frequently than G to A changes, but still provided a major source of these obviously inactivating mutations. (Fig. 5A). Frameshifts and other mutations were responsible for only a small proportion of protein-truncating mutations. Many of the C to T changes occurred in CG dinucleotides and are therefore likely to be caused by spontaneous post-integration deamination events. Notably, all of the stop codons that arose through the appearance of G to A mutations were due to a change from Trp (TGG) to termination codons (TAG, TGA or TAA), often reflecting the dinucleotide bias associated with the G to A changes observed in those species. The large number of stop codons generated at Trp codons, likely by APOBEC3-mediated cytidine deamination, would almost certainly functionally inactivate many of these endogenous retroviruses. Note that this analysis was confined to the CA-NTD sequences, and thus significantly underestimates the total number of termination codons in full-length proviruses.

In addition to stop codons, G to A and C to T changes introduced missense mutations in CA NTDs whose effects cannot be determined simply by inspecting sequence data. Although it was not practical to determine the effect of each G to A and C to T mutation in isolation, a few (fourteen) members of the libraries of chimeric CERV1- RhERV1b- or RhERV2-MLVs bore single, naturally occurring, missense mutations that could likely be attributed to enzymatic cytidine deamination (GG to AG changes) or spontaneous cytidine deamination (CG to CA or CG to TG changes). Chimeric MLVs bearing these single mutations were invariably less infectious than corresponding MLVs encoding the intact CERV1, RhERV1b or RhERV2 consensus CA-NTD sequence (Fig. 5B). The magnitude of decreases in infectivity were variable, and in one case a missense GG to AG mutation completely inactivated a CERV1-MLV chimera. Thus, amino acid differences from the consensus, likely attributable to enzymatic or spontaneous cytidine deamination decreased infectivity, suggesting that they were unlikely to be positively selected to provide benefit to a replicating virus. Rather, in addition to their propensity to induce the occurrence of termination codons, the abundant nonsynonymous G to A and C to T changes likely contributed significantly to a deleterious genetic load borne by these populations of endogenized proviruses.

## Discussion

Ancient epidemics of viral infection likely contributed to the selection of modern genes with potent antiretroviral activity, such as the TRIM5 and APOBEC3 families of antiretroviral factors. Since these selection events did not involve contemporary viruses, understanding how reciprocal selective pressures were imparted is problematic. However, the availability of nearly complete genome sequences for some modern primates, and the propensity of germline-tropic ancient retroviruses to be preserved in the genomes of modern organisms, potentially allows part or all of their sequences to be accessed and reconstituted in infectious form. In this study, we undertook an analysis of two groups of MLV-related retroviruses that are preserved in the genomes of modern old world primates, but are notably absent from human DNA. The goals of this study were to obtain evidence for or against the notion that TRIM5 and/or APOBEC3 limited the host range and/or contributed to the extinction of these retroviruses. Notably, a prototype gammaretrovirus, MLV, has been shown to be sensitive to inhibition by human APOBEC3G and one variant, N-MLV, has been shown to be sensitive to inhibition by several old world primate TRIM5α proteins. Thus, it was plausible that ancient gammaretroviruses might be relevant targets for host-range-limiting antiretroviral defense mechanisms.

By reconstituting infectious viruses that encoded endogenous retroviral CA-NTDs, we obtained in vitro evidence suggesting that TRIM5α proteins did not play a prominent role in the inactivation of, or limit the cross-species transmission of these primate gammaretroviruses. Indeed, all TRIM5 proteins tested lacked restriction activity against all endogenous N-terminal domain capsids studied, except in one instance where a RhERV2 CA-NTD carrying a E91K mutation was shown to be sensitive to chimpanzee TRIM5α. Nevertheless, a close relative of RhERV2, namely CERV2, is present in the chimpanzee genome. It is perhaps noteworthy that none of the CERV-2 CA-NTDs encode K91, perhaps suggesting that a limited subgroup of the CERV2/RhERV2 group of viruses had the propensity to colonize chimpanzees, or that chimpanzee TRIM5α might constrain CERV2 CA-NTD sequence variation.

There are two potential caveats to the conclusion that TRIM5α proteins did not cause extinction or impact the host range of these primate gammaretroviruses. First, the inactivation of these primate gammaretroviruses, and the consequent termination of the putative selective pressure imposed on TRIM5 genes, may have occurred in the sufficiently distant past that subsequent pressure on TRIM5 genes caused loss of gammaretroviral restriction activity. In other words, the modern TRIM5 proteins tested here may lack specificity for ancient viral capsids that ancestral TRIM5 proteins possessed. However, since the replication of CERV1/2 occurred after the divergence of humans and chimpanzees six million years ago, any significant shifts in restriction factor evolution caused by the rise and fall of species-specific retroviruses should be reflected in the divergence of the human and chimpanzee genes; but despite the absence of CERV1/2 from the human genome and its presence in the chimpanzee, TRIM5α remains largely conserved, in sequence and functional terms, in these two species. In short, the replication of CERV1/2 and their orthologs appears too recent relative to TRIM5 functional divergence for a subsequent shift in TRIM5α specificity to account for the observation that modern TRIM5α proteins do not inhibit these two groups of retroviruses.

Second, it is possible that there are determinants of TRIM sensitivity that are outside of the N-terminal domain of capsid. This was of particular concern given the findings of Kaiser et al. who, while this work was in progress, reported that a chimeric MLV encoding the p12 and capsid proteins of an ancestral CERV1, was indeed restricted by human and chimpanzee TRIM5α [31]. The presence of the CERV1 p12 domain could, theoretically, affect TRIM5 sensitivity, but all known determinants of TRIM5 or Fv1 sensitivity map to the CA-NTD [5]–[8],[33],[34]. Moreover, cleavage from the p12 domain is required for MLV CA to interact with TRIM5α and Fv1 [40], so it is difficult to see how p12 sequences could affect sensitivity to TRIM5α. More likely was the possibility that a CA-CTD element that affects capsid stability or TRIM5 interaction might affect TRIM5 sensitivity and would not be represented in our CERV1 CA-NTD/MLV chimera. However, when we generated a chimera that contained the full-length CERV1 CA, with either consensus or ancestral NTD linked to an ancestral CTD, the resulting virus was highly infectious (∼10^5^ IU/ml) and clearly insensitive to all TRIM5α proteins tested. Thus, this result supports the notion that CERV1 was not restricted by the TRIM5α proteins encountered in its natural target species and validates our experimental strategy for analyzing a broader range of CA-NTDs for TRIM5 sensitivity.

We note that TRIM5 is not the only TRIM family member that is a potential candidate for a suppressor of endogenous retroviral activity. TRIM1 in particular, as well as other TRIM proteins, have been demonstrated to exhibit anti-MLV activity in vitro [7],[41]. Additionally, other TRIM proteins, specifically TRIM22 can exhibit signatures of positive seletion, suggesting a potential role as an antiviral factor [42]. However, the identity or identities of viruses that were responsible for positive selection in TRIM genes remains to be determined. Lentiviruses, spumaretroviruses, and gammaretroviruses have all been shown to be intrinsically capable of being restricted by TRIM5 proteins [4]–[8],[43]. Thus retroviruses from any one, or any combination of these (or other) genra, could potentially have imposed selection on TRIM genes. Moroever, it is not necessarily the case that the retroviruses that were responsible for diversifying selection of TRIM5 are represented in the genomes of modern organinsms. Indeed, most retroviruses that infected ancestral hosts probably did not become established in the germ line of their host species.

While these findings do not suggest that TRIM5α proteins inhibited the replication of these two groups of primate gammaretroviruses, we did find clear evidence for encounters between the endogenized viruses and another major arm of the antiretroviral host defense, in both the chimpanzee and the rhesus macaque. This conclusion is based on the frequent occurrence of G to A changes that occurred in dinucleotide contexts (GG to AG and GA to AA) indicative of the action of APOBEC3 cytidine deaminases. Notably some viruses, in particular the rhesus macaque virus RhERV2, displayed two distinct dinucleotide biases on distinct proviruses, while only the GG to AG bias was evident in CERV-1 and CERV2. Thus, it appears that APOBE3G, the only known mutator that preferentially induces GG to AG changes, was largely responsible for hypermutation in chimpanzees while rhesus macaque gammaretroviruses were subjected to the action of two different APOPBEC3 proteins. Assays employing Vif-deficient HIV-1 indicate that rhesus macaque APOBEC3 proteins that induce GA to AA mutations, particularly APOBEC3F and APOBEC3H [14],[44],[45], are more active mutators relative to their human counterparts, while human APOBEC3G is a more potent mutator than rhesus macaque APOBEC3G [14],[45]. Thus, assuming that the properties of chimpanzee APOBEC3 proteins are similar to those of humans, the spectrum of mutations found in highly related gammaretroviruses that colonized two different primate species appears to correlate with the properties of the APOBEC3 proteins found therein.

Several analyses confirmed that the excessive G to A mutations in GG or GA dinucleotides were both nonrandom and not due to amplification of a relatively small number of G to A changes from founder viruses (data not shown). Moreover, by removing CG dinucleotides from our analyses, we confirmed that the GG to AG mutations observed were not due to spontaneous deamination at methylated CG dinucleotides. In some cases, the appearance of biased G to A mutations in CA-NTD was corroborated by the appearance of the same biases in linked Env or complete proviral sequences. Sometimes, however, individual CA-NTDs were not good predictors of frequent G to A mutation in other regions of the provirus. This is likely because: (i) the individual CA-NTDs are short (∼400 nt) sequences and thus subject to stochastic variation in the numbers of G to A mutations, (ii) variation in the mutation frequency (including 5′ to 3′ gradients) across an individual provirus, (iii) recombination between hypermutated and intact proviruses prior to or during deposition in the germline, and/or (iv) position-dependent intrinsic variation in non-APOBEC3 induced sequence diversity in different viral genes that may mask low frequency APOBEC3-mediated editing.

APOBEC3-mediated and spontaneous cytidine deamination events contributed a significant genetic burden to many of the individual proviruses analyzed here, through the frequent introduction of stop codons and other deleterious nonsynonomous mutations. Because of the high frequency of stop codons that were generated by GG to AG mutations, it is likely that APOBEC3 activity had an impact on the viral populations within each infected individual and may have inhibited intra- and interspecies transmissions. It is even possible that APOBEC3-induced mutation was responsible for terminating the replication of these retroviruses in their respective hosts. Such a definitive conclusion would be premature, however, because the frequency with which APOBEC3 mutated proviruses were deposited in the chimpanzee and rhesus macaque germ line may not be representative of the frequency with which APOBEC mutation actually occurred during active infection. Indeed, the population of proviruses present in the germ line is likely enriched for defective, including APOBEC3-mutated, variants, as these are less likely to be deleterious to the host. An intriguing possibility, currently under investigation, is that defective APOBEC-3 mutated proviruses may in fact be beneficial to the host as a source of defective interfering genomes or proteins.

Concurrently, we and others have found that another endogenous retrovirus, HERV-K, has also been subjected to hypermutation by APOBEC3G in humans [46],[47]. Additionally, some endogenous variants of MLV have been shown to bear similar footprints of encounters with the mouse APOBEC3 protein [32]. Thus, evidence is accumulating that APOBEC3 proteins, particularly APOBEC3G, provided a broad defense against a variety of formerly exogenous, now endogenous, retroviruses. Reconstitution of ancient retroviruses from remnants that have been preserved in the genomes of modern organisms offers the opportunity to probe and potentially explain the evolutionary history of host defenses against retroviruses.

## Materials and Methods

### Sequence retrieval

To identify MLV-related viruses in primate genomes, the amino acid sequence of the capsid N-terminal domain of moloney murine leukemia virus first was used in a TBLASTN search of the chimpanzee genome (http://www.ensembl.org). After retrieving all complete TBLASTN hits, the sequences were separated into two families, corresponding to CERV1 and CERV2, according to sequence homology and length. After removing redundant sequences, ClustalW was used to obtain nucleotide and amino acid consensus sequences for both CERV1 and CERV2 capsid N-terminal domains. The same method was used to find orthologues of CERV1 and CERV2 CA-NTDs in *Macaca mulatta* (RhERV1 and RhERV2, respectively). RhERV1 CA-NTDs included two phylogenetically distinct subgroups, termed RhERV1a and RhERV1b, and two independent consensus sequences were derived. Similarly, CA-NTD sequences from enMLV, were retrieved from the C57BL/6J genome sequence database. A phylogeny was reconstructed for all capsid NTDs using ClustalX1.8 and phylogenetic trees were drawn using Figtree.

Individual CA-NTD amino acid sequences were used in TBLASTN searches and regions positioned immediately 3′ to precise hits were inspected for Env-like sequences. The moloney MLV Env sequence was used to assist in the definition of theoretical CERV1/CERV2/RhERV1/RhERV2 Env open reading frames. To define a consensus envelope sequence for each endogenous retrovirus family, a single envelope amino acid sequence was used as a TBLASTN query. All resulting envelope sequences were aligned to derive a consensus sequence, regardless of their linkage to a previously retrieved capsid sequence. Analyses of the CA-linked envelope genes was then performed as for the CA-NTD sequences. In the case of RhERV2, two distinct groups of envelope sequences were observed and separate alignments and consensus sequences were derived for each subgroup. Similarly, three distinct enMLV envelopes were grouped according to the previously defined tropisms of these viruses: polytropic, modified polytropic, and xenotropic [48]. In the case of CERV1, because of the large numbers of unique CA-NTD sequences, the analysis was confined to two groups of 10 CA-NTDs that were each used to define linked Env sequences. One CA-NTD group had zero or one G to A mutations, and a second group had ≥4 G to A mutations.

Because of the small number of (10) CERV2 integrations in the chimpanzee germ line, all CERV2 proviral sequences were compiled. Flanking regions 1500 nucleotides 5′ and 8000 nucleotides 3′ to the capsid NTDs were aligned. Four of the proviruses aligned for 8293 nucleotides with pairwise identity scores of at least 83%. Therefore, those portions of sequence were trimmed and defined as full length CERV2 provirus. Six additional incomplete proviruses were defined. One database derived proviral sequence was excluded because a 99.9% sequence identity with another locus made it unlikely to be an independent retroviral integration, and is instead likely to be either a genomic duplication or an error in the compiling of genomic sequence.

### Generation and functional analysis of endogenous retrovirus CA-NTD/MLV chimera libraries

A library of chimeric GagPol plasmids was generated that contained MoMLV GagPol in which the capsid NTD was replaced with that from endogenous retroviruses. Genomic DNA was isolated using standard protocols from *Pan troglodytes verus* skin fibroblasts AG06939 (Coriell), *Macaca mulatta* 221 cells, and NIH 3T3 cells and used as PCR template. Primers were designed to anneal to the capsid NTD consensus sequences described above. Because of relatively high conservation at the 3′ end of the capsid NTD, the same reverse primer was used for all endogenous retrovirus families: 5′- TACYTTRGCCAAATTRGTRGG -3′. The 5′ primers were specific to each virus family and all contained a BsmI restriction site: CERV1: 5′- CTCGCAGGCATTCCCCCTTCGGGAAATAGG -3′; CERV2: 5′- CTCGCAGGCATTCCCCCTCCGCACCGTG -3′. To amplify capsid genes from the Rhesus macaque and mouse genomes, primers were designed to anneal to a p12 region immediately 5′ of the capsid- RERV1a: 5′- CAGCYGCCTGACTCYAYGGTGGCATTCCCCCTT -3′; RERV1b: 5′- CRACTCCCTGACTCCACYGTGGCATTCCCTCTC -3′; RERV2: 5′- CCTTCYACTTGGCAATCCTCGGCATTCCCCCTC -3′; and enMLV: 5′- GCRGAYTCCACCWCCTCYCRGGCATTCCCACTC -3′. A small fragment was amplified from MLV GagPol using the reverse complement of the capsid NTD 3′ primer: 5′- CCYACYAATTTGGCYAARGTA -3′; and a primer annealing in the MLV capsid C-terminal domain: 5′- CTTCTAACCTCTCTAACTTTCTCC -3′. The amplified portion of the MLV GagPol plasmid was engineered to contain an AfeI restriction site. Thereafter, an overlapping PCR product was generated that included the amplified endogenous virus capsid NTDs and the MoMLV capsid CTD fragment. This PCR product was cloned into the MLV GagPol plasmid using BsmI and AfeI restriction enzymes. The resulting chimeric GagPol plasmids were isolated and screened for functional capsid genes by cotransfection with an MLV-based vector containing GFP and VSV-G envelope using polyethylenimine (PEI) in 293T cells. Supernatant from these cells were used to infect hamster CHO-KI-derived cells. Two days post-infection, cells were trypsinized, fixed in 2% PFA and subjected to FACS analysis using a Guava Easycyte to determine the percentage infected (GFP-positive) cells. To measure TRIM5α, TRIMCyp and Fv1 sensitivity, Mus dunni tail fibroblasts (MDTF) or MDTF stably expressing human TRIM5α, chimpanzee TRIM5α, rhesus macaque TRIM5α, african green monkey TRIM5α, owl monkey TRIMCyp, Fv1^N^, Fv1^B^, or Fv1^NB^ that have previously been described [5],[49],[50]. Fv1^NB^ is an unnatural chimeric Fv1 protein that restricts both N-tropic and B-tropic MLV strains. The TRIM5 and Fv1-expressing cells were infected in the presence of 5 µg/mL polybrene and GFP-positive cells were quantified by FACS analysis two days post-infection.

To determine whether the E91K mutation in the RhERV2 CA-NTD modulates its sensitivity to human and chimpanzee TRIM5α, this point mutation was introduced by PCR into a chimeric MLV GagPol plasmid containing a consensus RhERV2 CA-NTD sequence. Additionally, to determine whether the CA-CTD altered CERV1 sensitivity to TRIM5α, a CERV1 CTD sequence, as described in Kaiser S.M. et. al. [31], was synthesized using a series of overlapping ∼60 nucleotide olignucleotides. The resulting product was then used in an overlapping PCR reaction with the CERV1 CA-NTD consensus sequence and this full-length capsid was cloned into the MLV GagPol plasmid. In order to match the CERV1 CA-NTD consensus to the ancestral sequence described in Kaiser et. al. [31], the R35Q mutation was also introduced using PCR-based mutagenesis into this CERV1 full-length capsid/MLV chimera.

### Sequence analysis

Mutations and their dinucleotide sequence contexts were quantified using Hypermut (http://www.hiv.lanl.gov/content/sequence/HYPERMUT/hypermut.html). We generated fasta alignments using MacVector to use as input for Hypermut and the consensus sequence for each virus family (described above) was used as a reference sequence. The Hypermut output provided the total number nucleotide changes (each insertion/deletion was reduced to a single nucleotide change in MacVector) as well as the number of G to A mutations relative to the consensus sequence. Hypermut also provided the number of G to A changes that occurred in each of the four possible dinucleotide contexts when the nucleotide immediately 3′ to the G (or the +1 position) in the consensus sequence is considered (i.e. GG, GA, GC and GT). To quantify the −1 position for C to T changes, the +1 position for C to T changes, and the −1 position for G to A changes, the reverse complement, complement, and reverse of each alignment was generated respectively and used as Hypermut inputs. The latter two analyses allowed us to quantify the number of C to T and G to A changes occurring in CG dinucleotides. We repeated this analysis, after removal of G to A and C to T changes occurring in CG dinucleotides. In some charts, the percentages of G to A or C to T changes that were in each dinucleotide context were normalized according to the dinucleotide composition of a given consensus sequence. P-values were calculated for these data using a chi-square goodness of fit statistical test.

## Supporting Information

Dataset S1 (0.03 MB PDF)

Figure S1 Phylogenetic tree illustrating the relationship between endogenous primate gammaretroviral Env sequences and other gammaretroviral Envs. A sequence alignment, and phylogeny of Env sequences from CERV1, CERV2, RhERV1a, RhERV1b, RhERV2, and enMLV and various prototype gammaretroviruses was generated using ClustalX software. The tree diagram was generated and edited using FigTree software.
(0.39 MB TIF)

Figure S2 Examples of the RhERV2 proviruses illustrating variation in type, burden, and distribution of G to A mutations among endogenous primate gammaretroviruses. Each horizontal line represents a complete or nearly complete ∼8.5 kB provirus (nucleotide position scale is given at the bottom of the diagram), vertical marks indicate the position of G to A mutations relative to the RhERV2 consensus sequence and are color-coded according to dinucleotide context (ie the nucleotide in the +1 position relative to each G to A change; red = GG to AG, cyan = GA to AA, green = GC to AC, magenta = GT to AT).
(0.44 MB TIF)

Figure S3 Comparative analysis of the context in which G to A versus C to T changes occur in primate gammaretroviral CA-NTDs. (A) Each sequence is plotted as a bar graph (one bar for each provirus) and color-coded according to dinucleotide context (ie the nucleotide in the +1 position relative to each G to A change; red = GG to AG, Cyan = GA to AA, Green = GC to AC, magenta = GT to AT). The CA NTD sequences are arranged from left to right in order of increasing numbers of total G to A changes in CA-NTD sequences (panel A contains the same data as the upper panels of Fig. 4A) (B) Same analysis as in A, except mutations were enumerated after removal of minus strand CG to TG (plus strand CG to CA) mutations. (C) Analysis of plus strand C to T mutations, in the same CA-NTD sequences, in the same order, (left to right) as in A and B. C to T changes are color-coded according to the nucleotide in the −1 position relative to each C to T change; red = CC to CT, Cyan = TC to TT, Green = GC to GT, magenta = AC to AT) (D) Same analysis as in C, except mutations were enumerated after removal of plus strand CG to TG mutations.
(0.46 MB TIF)

Figure S4 Comparative analysis of the burden of, and the context in which, G to A versus C to T changes occur in endogenous primate and murine gammaretroviral Env sequences. (A) Each sequence is plotted as a bar graph (one bar for each provirus) and color-coded according to dinucleotide context as in Fig. S2. The Env sequences are derived from the same proviruses as the CA-NTD sequences shown in Fig. S3, and arranged from left to right in the same order (panel A contains the same data as the lower panels of Fig. 4A) (B) Same analysis as in A, except mutations were enumerated after removal of minus strand CG to TG (plus strand CG to CA) mutations. (C) Analysis of plus strand C to T mutations, in the same Env sequences, in the same order, (left to right) as in A and B. C to T changes are color-coded according to the nucleotide in the −1 position relative to each C to T change as is Fig. S1. (D) Same analysis as in C, except mutations were enumerated after removal of plus strand CG to TG mutations.
(0.46 MB TIF)

Figure S5 Comparative analysis of the burden of, and the context in which, G to A versus C to T changes occur in CERV2 proviruses. (A) Each sequence is plotted as a bar graph (one bar for each provirus) and color-coded according to the dinucleotide context in which G to A mutations occur, as in Fig. S3. The proviral sequences correspond to the CA-NTD sequences shown in Fig. S3, and arranged from left to right in the same order. The left panel shows analysis without removal of minus strand CG dinucleotides, while the right panel shows analysis after their removal. (B) Analysis of plus strand C to T mutations, in the same CERV2 proviral sequences, in the same order, (left to right). C to T changes are color-coded according to the nucleotide in the −1 position relative to each C to T change as is Fig. S2. The left panel shows analysis without removal of plus strand CG dinucleotides, while the right panel shows analysis after their removal.
(0.18 MB TIF)
