# Supplementary material for: Evidence for Restriction of Ancient Primate Gammaretroviruses by APOBEC3 but Not TRIM5α Proteins
Source: PLoS Pathog. 2008 Oct 17;4(10):e1000181. doi: 10.1371/journal.ppat.1000181 (PMC2564838; doi:10.1371/journal.ppat.1000181)
Supplement: Dataset S1 — (0.03 MB PDF) [file ppat.1000181.s001.pdf]

Dataset S1

CA-NTD amino acid sequences

CERV1 (consensus)

PLREIGPPDDTGIPRLQYWPFSTSDLYNWKTQSARFSDNPKDLLALLDSVMFTHQPT WDDCQQLLRILFTTEERERIQIEARKLVPGDDGQPTANPDLINATFPLTRPAWDYNT AEGRGRLHLYRQTLMAGLRAAARK

CERV1#23

PLREIGPPDDTGIPRLQYWPFSTSDLYNWKTQSARFSDNPKDLLALLDSVMFTHQPT WDDCQQLLRILFTTEERERIQIEARKLVPGDDGQPTANPDLINATFPLTRPAWDYNT AEGRGQLHLYRQTLMAGLRAAARK

CERV1#34

PLREIGPPDDTGIPRLQYWPFSTSDLYNWKTQSARFSDNPKDLLALLDSVMFTHQPT WDDCQQLLRILFTTEERERIQIEARKLVPGDDSQPTANPDLINATFPLTRPAWDYNTA EGRGRLHLYRQTLMAGLRAAARK

CERV1#46

PLREIGPPDDTGIPRLQYWPFSTSDLYNWKTQSARFSDNPKDLLALLDSVMFTHQPT WDDCQQLLRILFTTEERERIQIEARKLVPEDDGQPTANPDLINATFPLTRPAWDYNTA EGRGRLHLYRQTLMAGLRAAARK

CERV1#137

PLREIGPPDDTGIPRLQYWPFSTSDLYNWKTQSARFSDNPKDLLALLDSVMFTHQPT WDDCQQLLRILFTTEERERIQIEARKLVPGDDGQPTANPDLINATFPLTRPAWDYNM AEGRGRLHLYRQTLMAGLRAAARK

CERV1#319

PLREIGPPDDTGIPRLQYWPFSTSDLYNWKSQSARFSDNPKDLLALLDSVMFTHQPT WDDCQQLLRILFTTEERERIQIEARKLVLGDDGQPTANPDLINATFPLTRPAWDYNT AEGRGRLHLYRQTLMAGLRAAARK

RhERV1a (consensus)

**PLREIGPPDDTGIPRLQYWPFSTSDLYNWKTQSARFSDNPKDLLALLDSVMFTHQPT
WDDCQQLLRILFTTEERERIQVEARKLVPGDDGQPTANPDLINATFPLTRPAWDYNT
AEGRGRLHLYRQTLMAGLRAAARK**

RhERV1a#168

**PLREIGPPDDTGIPRLQYWPFSTSDLYNWKTQSARFSDNPKDLLSLLDSVMFTHQPT
WDDCQQLLRILFTTEERERIQVEARKLVPGDDGQPTANPDLINATFPLTRPAWDYNT
AEGRGRLRLYRQTLMAGLRAAARK**

RhERV1a#181

**PLREIGPPDDTGIPRLQYWPFSTSDLYNWKTQSARFSDNPKDLLALLDSVMFTHQPT
WDDCQQLLRILFTTEERERIQVEARKLVLGDDGQPTANPDLINATFPLTRPAWDYNT
AEGRGRLCLYRQTLMAGLRAAARK**

RhERV1b (consensus)

**PLREIGSLDDTGLSRLMYWPFSTSDLYNWKSQNARFSDNPKDLTSLLDSVMFTHQPT
WDDCQQLLRILFTTEERERIQVEARKLVPGDDGQPTANPDLINAAFPLTRPRWDYNT
AEGRGRLLIYRQTLMAGLRAAARK**

RhERV1b#67

**PLREIGSLDDTGLSRLMYWPFSTSDLYNWKSQNARFSDNPKDLTLLLDSVMFTHQPT
WDDCQQLLRILFTTEERERIQVEARKLVPGDDGQPTANPDLINAAFPLTRPRWDYNT
AEGRGRLLIYRQTLMAGLRAAARK**

RhERV1b#75

**PLREIGSLDDTGLSRLMYWPFSTSDLYNWKSQNARFSDNPKDLTSLLDSVMFTHQPT
WDDCQQLLRILFTTEERERIQVEARKLVPGDDGQPTANPDLINVAFPLTRPRWDYNT
AEGRGRLLIYRQTLMAGLRAAARK**

RhERV1b#77

**PLREIGSLDDTGLSRLMYWPFSTSDLYNWKSQNARFSDNPKDLTSLLDSVMFTHQPT
WDDCQQLLRILFTTEERERIQVEARKLVPGDDGQPTANPDLINAAFPLTRPRWDYN
MAEGRGRLLIYRQTLMAGLRAAARK**

RhERV1b#92

PLREIGSFDDTGLSRLMYWPFSTSDLYN*KSQNARFSDNPKDLTSLLDSVMFTHQPT
WDDCQQLLRILFTTEERERIQVEARKLVPGDDGQPTANPDLINAAFPLTRPRWDYNT
AEGRGRLLIYRQTLMAGLRAAARK

CERV2 (consensus)

PLRTVNRTVQYWPFSASDLYNWKTHNPPFSQDPQALTALIESILLTHQPTWDDCQQ
LLQVLLTTEERQRVLLEARKHVPGPGGLPTQLPNEIDEGFPLTRPDWDYETASGRESLR
IYRQALLAGLKGAGKR

CERV2#32

PLRTVNRTVQYWPFSASDLYNWKTHNPPFSQDPQALTSLIESILLTHQPTWDDCQQ
LLQVLLTTEERQRVLLEARKNVPGPGGLPTQLPNEIDEGFPLTRLDWDYETATGRESLR
IYRQALLAGLKGAGKR

RhERV2 (consensus)

PLRTVNRTVQYWPFSASDLYNWKTHNPSFSQDPQALTSLIESILLTHQPTWDDCQQ
LLQVLLTTEERQRVLLEARKNVPGPGGLPTQLPNEIDEGFPLTRPDWDYETAPGRESLR
IYRQALLAGLKGAGKR

RhERV2#53

PLRTVNRTVQYWPFSASDLYNWKTHNPSFSQDPQALTSLIESILLTHQPTWDDCQQ
LLQVLLTTEERQRVLLEARKNVPGPGGLPTQLPNEIDEGFPLTRPDWDYETAPGRESLR
IYCQALLAGLKGAGKR

RhERV2#55

PLRTVNRTVQYWPFSASDLYNWKTHNPSFSQDPQALTSLIESILLTHQPTWDDCQQ
LLQVLLTTEERQRVLLEARKNVPGPGGLPTQLPNEIDEGFPLTRPDWDYEMAPGRESL
RIYRQALLAGLKGAGKR

RhERV2#58

PLRTVNRTVQYWPFSASDLYNWKTHNPSFSQDPQALTSLIESILLTHQPTWDDCQQ
LLQVLLTTEERQQVLLEARKNVPGPGGLPTQLPNKIDEGFPLTRPDWDYETAPGKESL
QIYRQALLAGLKGAGKR

RhERV2#63

PLRTVNRTVQYWPFSASDLYNWKTHNPSFSQDPQALTSLIESILLTHQPTWDDCQQ
LLQVLLTTEERQRVLLEARKNVPGPGGLPTQLPNEIDEGFPLTRPDWDYETAPGRESLR
IYRQALLAGLKGAGKC

RhERV2#76

PLRTVNRTVQYWPFSASDLYNWKTHNPSFSQDPQALTSLIESILLTHQPTWDDCQQ
LLQVLLTTEERQRVLLEARKNVPGPGGLPTQLPNEIDEGFPLTRPDWDYETAPGRESLR
IYHQALLAGLKGAGKR

RhERV2#91

PLRTVNRTVQYWPFSASDLYNWKTHNPSFSQDPQALTSLIESILLTHQPTWDDCQQ
LLQVLLTTEERQRVLLEARKNVPGPGGLPTQLPNEIDEGFPLTRPDWDYETAPGRESLR
IYRQALLAGLKGAGKH

RhERV2#98

PLRTVNRTVQYWPFSASDLYNWKTHNPSFSQDPQALTSLIESILLTHQPTWDDCQQ
LLQVLLTTEERQRVLLEARKNVPGPGGLPTQLPNEIDEGFPLTHPDWDYETAPGRESL
RIYRQALLAGLKGAGKR

RhERV2 E91K

PLRTVNRTVQYWPFSASDLYNWKTHNPSFSQDPQALTSLIESILLTHQPTWDDCQQ
LLQVLLTTEERQRVLLEARKNVPGPGGLPTQLPNKIDEGFPLTRPDWDYETAPGRESL
RIYRQALLAGLKGAGKR

enMLV(consensus)

PLRMGGDGQLQYWPFSSSDLYNWKNNNPSFSEDPGKLTALIESVLITHQPTWDDC
QQLLGTLLTGEEKQRVLLEARKAVRGNDGRPTQLPNEVNAAFPLERPDWDYTTTEGR
NHLVLYRQLLLAGLQNAGRS

enMLV#109

PLRMGGDGQLQYWPFSSSDLYNWKNNNPSFSEDPGKLTALIESVLITHQPTWDDC
QQLLGTLLTGEEKQRVLLEARKAVRGNDGRPTQLPNEINAAFPLERPDWDYTTTEGR
NHLVLYRQLLLAGLQNAGRS

enMLV#111

PLRMGGDGQLQYWPFSSSDLYNWKNNNPSFSEDPGKLTALIESVLITHQPTWDDC
QQLLGTLLTGEEKQRVLLEARKAVRGNDGRPTQLPNEVNAAFPLERPGWDYTTTEGR
NHLVLYRQLLLAGLQNAGRS

enMLV#116

PLRMGGDGQLQYWPFSSSDLYNWKNNNPPFSEDPGKLTALIESVLITHQPTWDDC
QQLLGTLLTGEEKQRVLLEARKAVRGNDGRPTQLPNEINAAFPLERPDWDYTTTEGR
NHLVLYRQLLLAGLQNAGRS

enMLV#124

PLRMGRDGQLQYWPFSSSDLYNWKNNNPSFSEDPGKLTALIESVLITHQPTWDDCQ
QLLGTLLTGEEKQRVLLEARKAVRGNDGRPTQLPNEVNAAFPLERPGWDYTTTEGRN
HLVLYRQLLLAGLQNAGRS

enMLV#127

PLRMGGDGQLQYWPFSSSDLYNWKNNNPSFSEDPGKLTALIESVLITHQPTWDDC
QQLLGTLLTGEEKQRVLLEARKAVRGDDGRPTQLPNEVNAAFPLERPGWDYTTTEGR
NHLVLYRQLLLAGLQNAGRS

Complete CA sequences

CERV1(consensus)

PLREIGPPDDTGIPRLQYWPFSTSDLYNWKTQSARFSDNPKDLLALLDSVMFTHQPT
WDDCQQLLRILFTTEERERIQIEARKLVPGDDGQPTANPDLINATFPLTRPAWDYNT
AEGRGRLHLYRQTLMAGLRAAARKPTNLAKVYSILQGKTESPATYLERLMEAFRQYTP
IDPEAPGSQAAVVMSFVNQAAPDIKRKLQKLEDLEGKRIQDLLQIAQRVYNNRDTPE
EKQFKATEKMTKVL

CERV1(ancestral)

PLREIGPPDDTGIPRLQYWPFSTSDLYNWKTQSAQFSDNPKDLLALLDSVMFTHQPT
WDDCQQLLRILFTTEERERIQIEARKLVPGDDGQPTANPDLINATFPLTRPAWDYNT
AEGRGRLHLYRQTLMAGLRAAARKPTNLAKVYSILQGKTESPATYLERLMEAFRQYTP
IDPEAPGSQAAVVMSFVNQAAPDIKRKLQKLEDLEGKRIQDLLQIAQRVYNNRDTPE
EKQFKATEKMTKVL
