# Supplementary figures and images for: Evidence for Restriction of Ancient Primate Gammaretroviruses by APOBEC3 but Not TRIM5α Proteins
Source: PLoS Pathog. 2008 Oct 17;4(10):e1000181. doi: 10.1371/journal.ppat.1000181 (PMC2564838; doi:10.1371/journal.ppat.1000181)

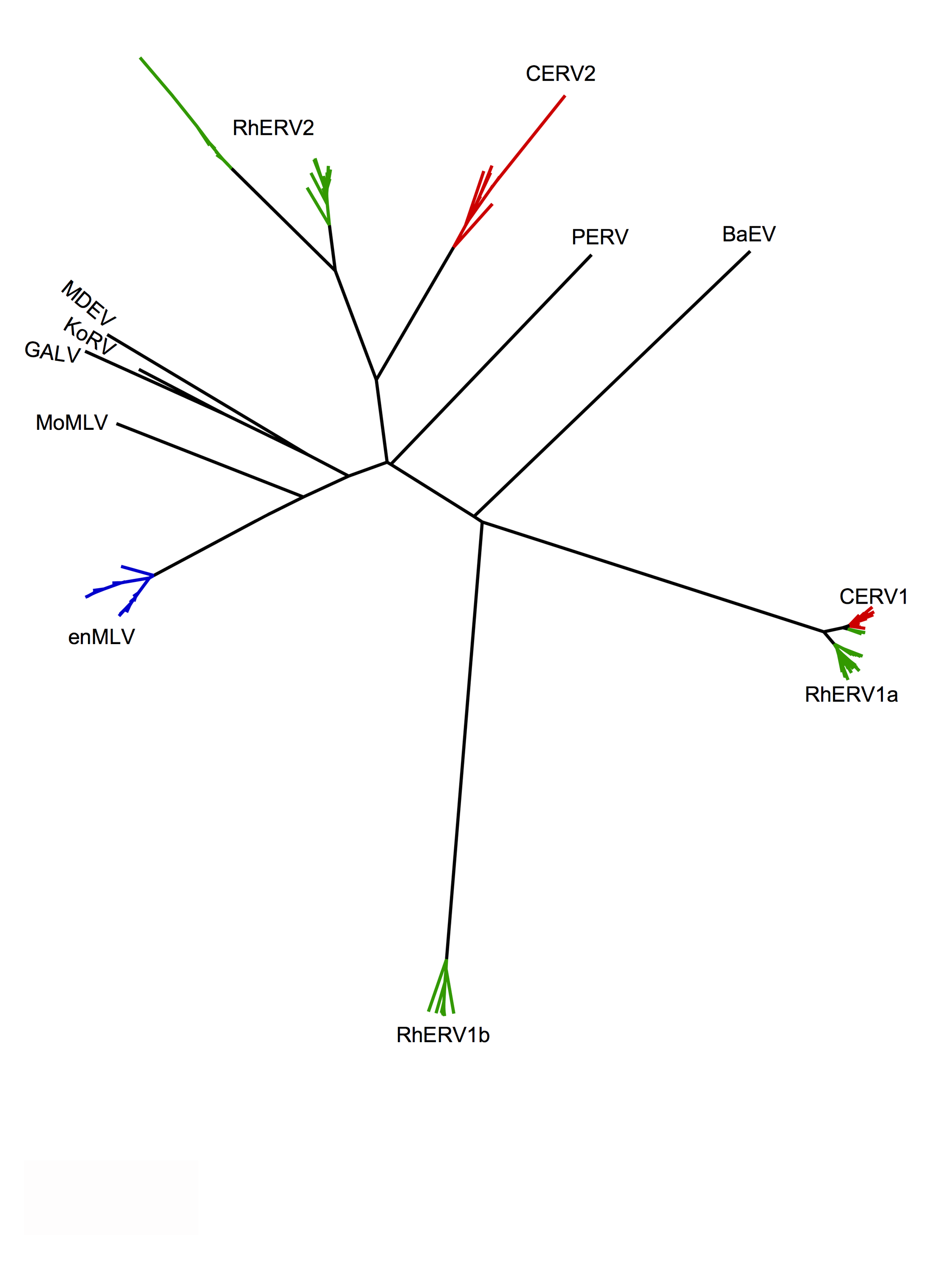

Supplement: Figure S1 — Phylogenetic tree illustrating the relationship between endogenous primate gammaretroviral Env sequences and other gammaretroviral Envs. A sequence alignment, and phylogeny of Env sequences from CERV1, CERV2, RhERV1a, RhERV1b, RhERV2, and enMLV and various prototype gammaretroviruses was generated using ClustalX software. The tree diagram was generated and edited using FigTree software. (0.39 MB TIF) [file ppat.1000181.s002.tif]

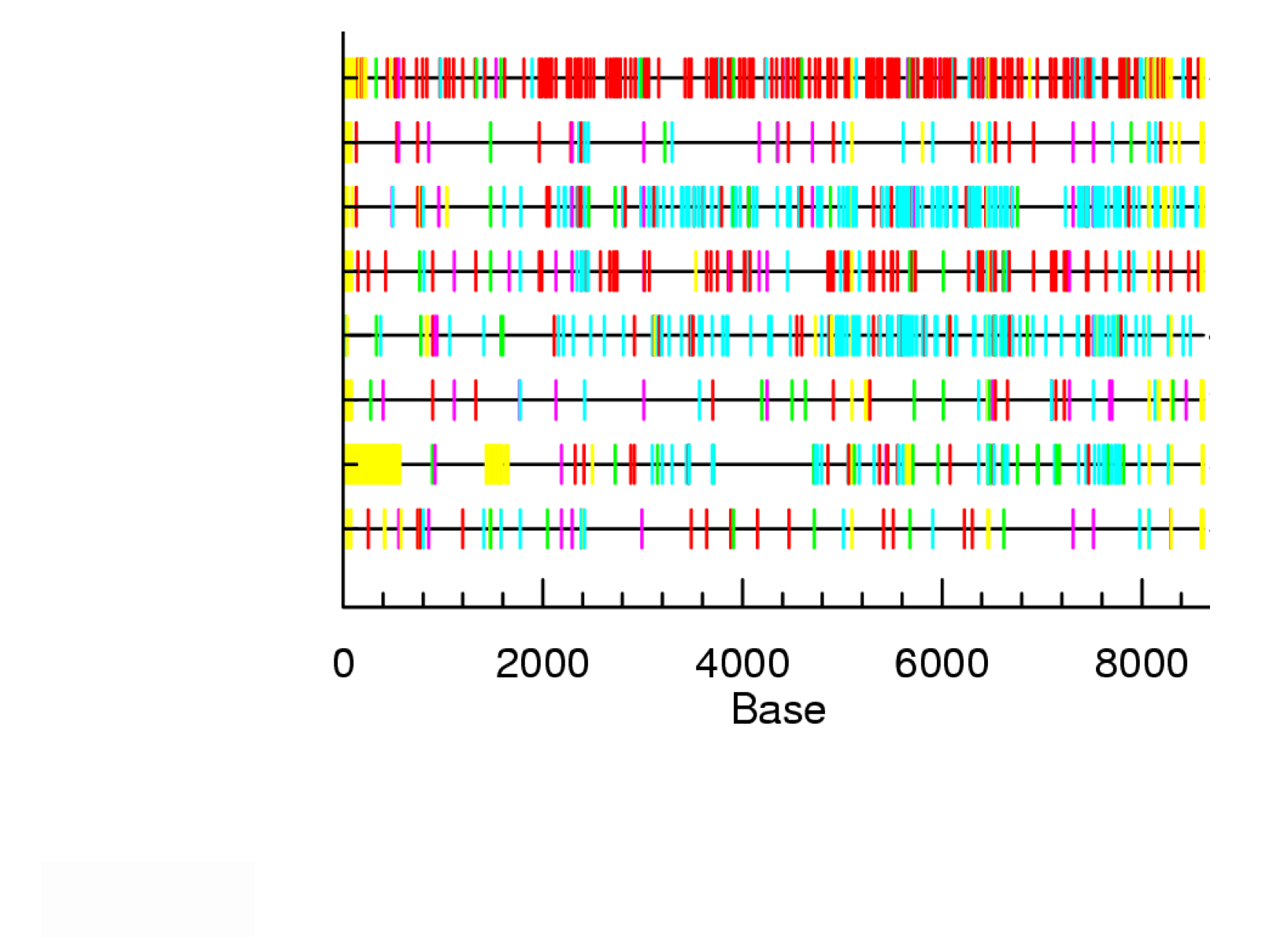

Supplement: Figure S2 — Examples of the RhERV2 proviruses illustrating variation in type, burden, and distribution of G to A mutations among endogenous primate gammaretroviruses. Each horizontal line represents a complete or nearly complete ∼8.5 kB provirus (nucleotide position scale is given at the bottom of the diagram), vertical marks indicate the position of G to A mutations relative to the RhERV2 consensus sequence and are color-coded according to dinucleotide context (ie the nucleotide in the +1 position relative to each G to A change; red = GG to AG, cyan = GA to AA, green = GC to AC, magenta = GT to AT). (0.44 MB TIF) [file ppat.1000181.s003.tif]

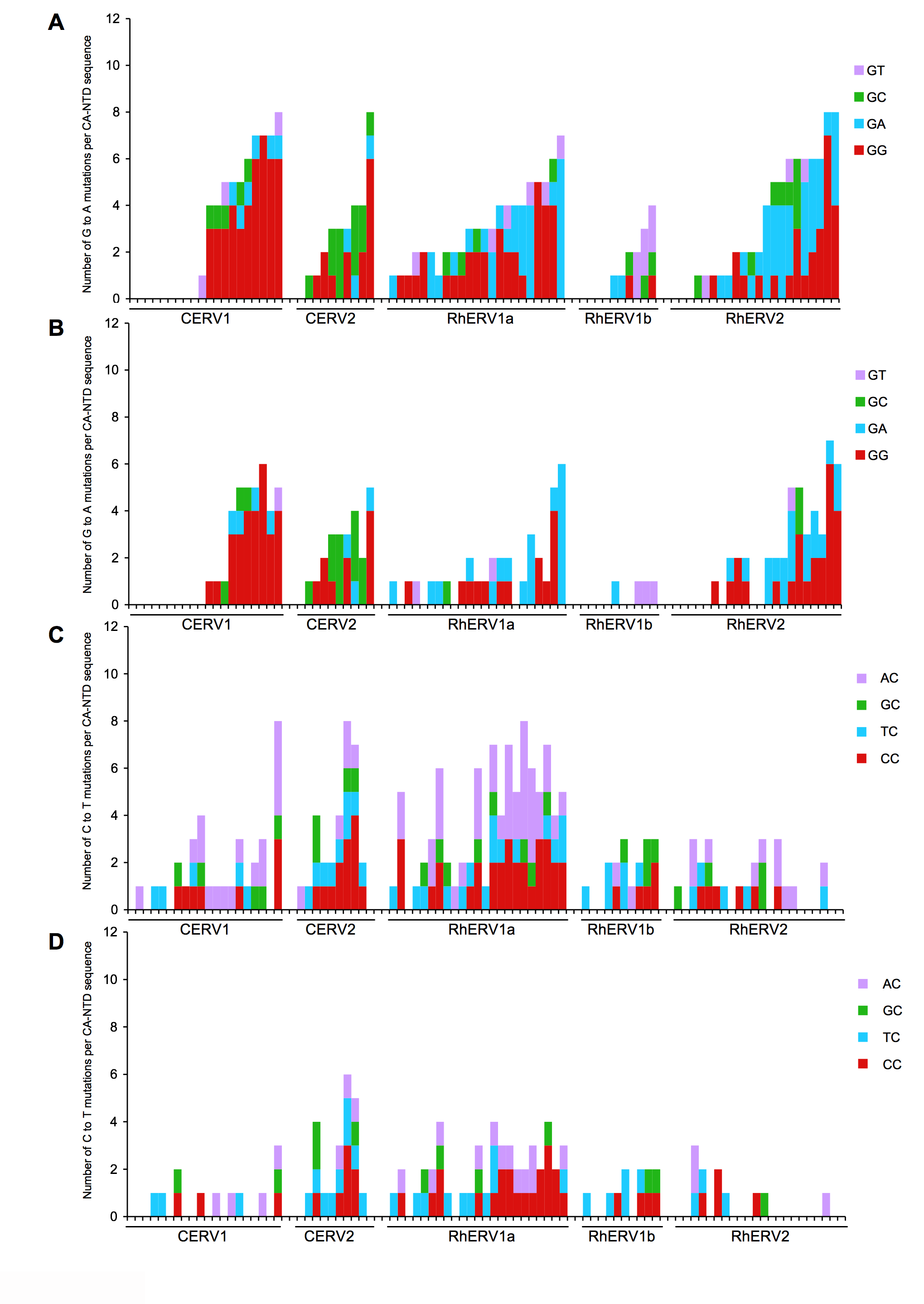

Supplement: Figure S3 — Comparative analysis of the context in which G to A versus C to T changes occur in primate gammaretroviral CA-NTDs. (A) Each sequence is plotted as a bar graph (one bar for each provirus) and color-coded according to dinucleotide context (ie the nucleotide in the +1 position relative to each G to A change; red = GG to AG, Cyan = GA to AA, Green = GC to AC, magenta = GT to AT). The CA NTD sequences are arranged from left to right in order of increasing numbers of total G to A changes in CA-NTD sequences (panel A contains the same data as the upper panels of Fig. 4A) (B) Same analysis as in A, except mutations were enumerated after removal of minus strand CG to TG (plus strand CG to CA) mutations. (C) Analysis of plus strand C to T mutations, in the same CA-NTD sequences, in the same order, (left to right) as in A and B. C to T changes are color-coded according to the nucleotide in the −1 position relative to each C to T change; red = CC to CT, Cyan = TC to TT, Green = GC to GT, magenta = AC to AT) (D) Same analysis as in C, except mutations were enumerated after removal of plus strand CG to TG mutations. (0.46 MB TIF) [file ppat.1000181.s004.tif]

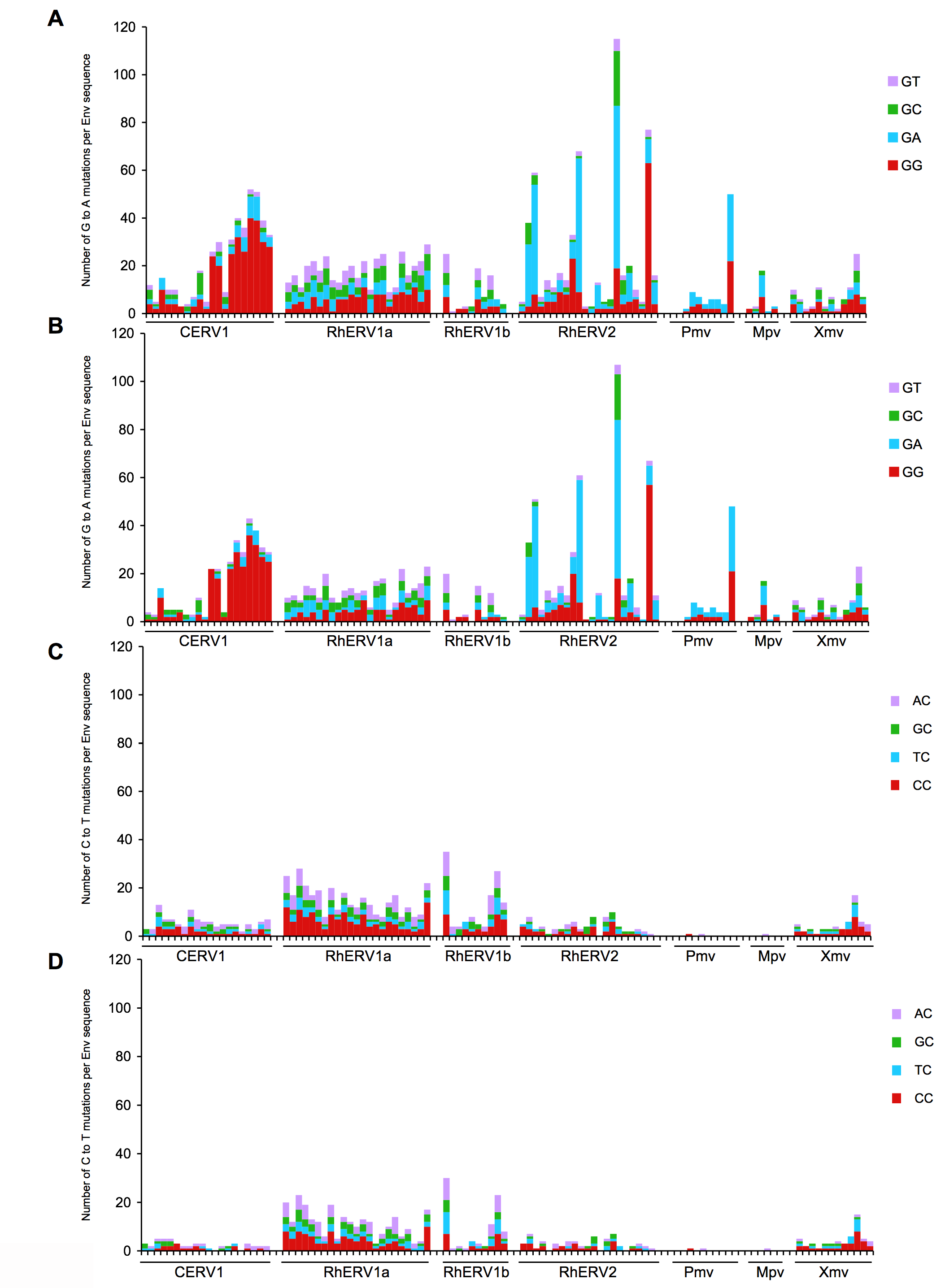

Supplement: Figure S4 — Comparative analysis of the burden of, and the context in which, G to A versus C to T changes occur in endogenous primate and murine gammaretroviral Env sequences. (A) Each sequence is plotted as a bar graph (one bar for each provirus) and color-coded according to dinucleotide context as in Fig. S2. The Env sequences are derived from the same proviruses as the CA-NTD sequences shown in Fig. S3, and arranged from left to right in the same order (panel A contains the same data as the lower panels of Fig. 4A) (B) Same analysis as in A, except mutations were enumerated after removal of minus strand CG to TG (plus strand CG to CA) mutations. (C) Analysis of plus strand C to T mutations, in the same Env sequences, in the same order, (left to right) as in A and B. C to T changes are color-coded according to the nucleotide in the −1 position relative to each C to T change as is Fig. S1. (D) Same analysis as in C, except mutations were enumerated after removal of plus strand CG to TG mutations. (0.46 MB TIF) [file ppat.1000181.s005.tif]

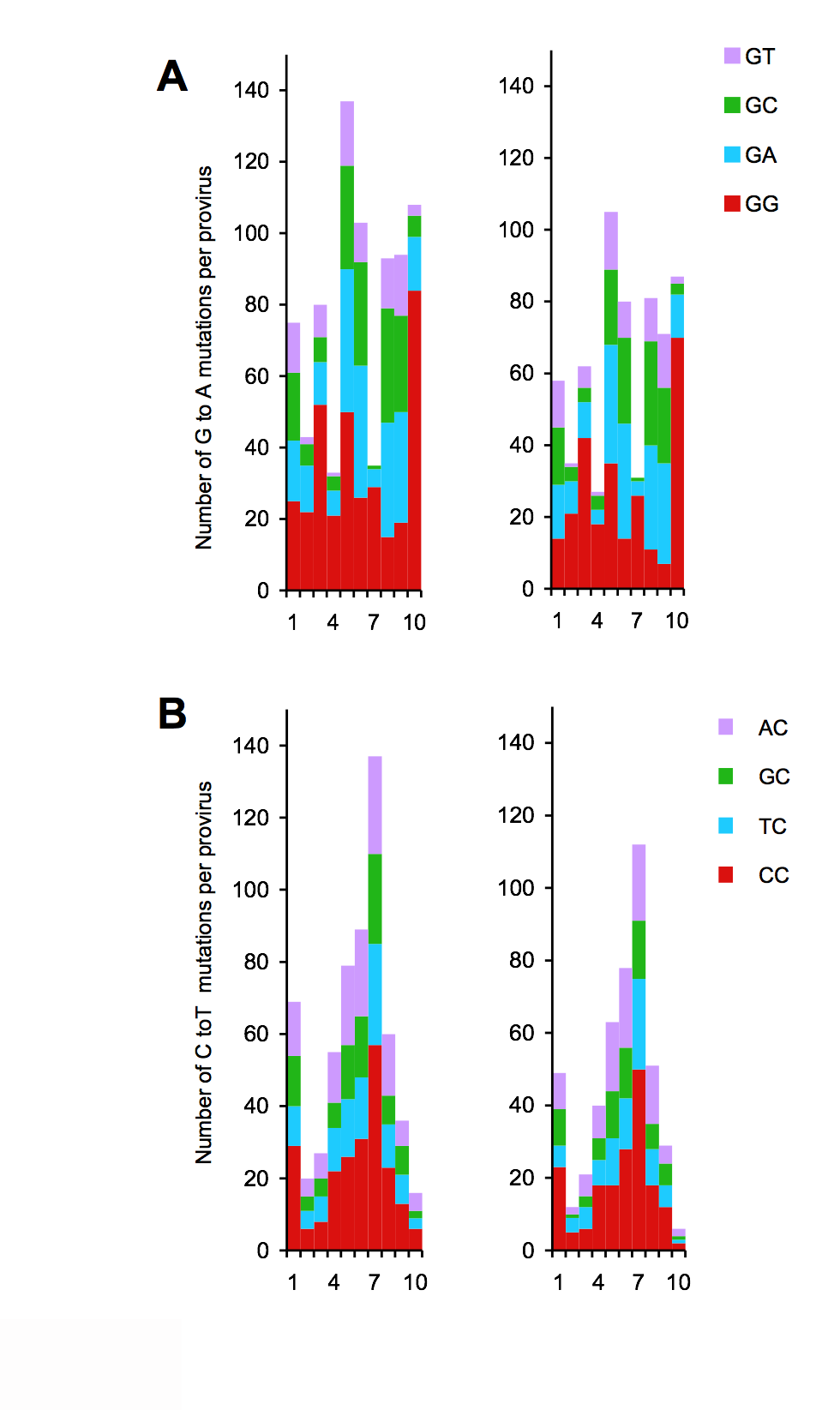

Supplement: Figure S5 — Comparative analysis of the burden of, and the context in which, G to A versus C to T changes occur in CERV2 proviruses. (A) Each sequence is plotted as a bar graph (one bar for each provirus) and color-coded according to the dinucleotide context in which G to A mutations occur, as in Fig. S3. The proviral sequences correspond to the CA-NTD sequences shown in Fig. S3, and arranged from left to right in the same order. The left panel shows analysis without removal of minus strand CG dinucleotides, while the right panel shows analysis after their removal. (B) Analysis of plus strand C to T mutations, in the same CERV2 proviral sequences, in the same order, (left to right). C to T changes are color-coded according to the nucleotide in the −1 position relative to each C to T change as is Fig. S2. The left panel shows analysis without removal of plus strand CG dinucleotides, while the right panel shows analysis after their removal. (0.18 MB TIF) [file ppat.1000181.s006.tif]
